# Crashworthiness Investigations for 3D-Printed Multi-Layer Multi-Topology Engineering Resin Lattice Materials

**DOI:** 10.3390/ma17194844

**Published:** 2024-09-30

**Authors:** Autumn R. Bernard, Muhammet Muaz Yalçın, Mostafa S. A. ElSayed

**Affiliations:** 1 Department of Mechanical and Aerospace Engineering, Carleton University, Ottawa, ON K1S 5B6, Canada; autumnbernard@cmail.carleton.ca (A.R.B.); mostafaelsayed@cunet.carleton.ca (M.S.A.E.); 2 Department of Mechanical Engineering, Sakarya University, 54050 Serdivan, Turkey

**Keywords:** cellular materials, cubic lattice topology, energy absorption performance, experimental techniques, octet lattice topology, stereolithography

## Abstract

In comparison to monolithic materials, cellular solids have superior energy absorption capabilities. Of particular interest within this category are the periodic lattice materials, which offer repeatable and highly customizable behavior, particularly in combination with advances in additive manufacturing technologies. In this paper, the crashworthiness of engineering multi-layer, multi-topology (MLMT) resin lattices is experimentally examined. First, the response of a single- and three-layer single topology cubic and octet lattices, at a relative density of 30%, is investigated. Then, the response of MLMT lattices is characterized and compared to those single-topology lattices. Crashworthiness data were collected for all topology arrangements, finding that while the three-layer cubic and octet lattices were capable of absorbing 9.8 J and 7.8 J, respectively, up to their respective densification points, the unique MLMT lattices were capable of absorbing more: 19.0 J (octet-cube-octet) and 22.4 J (cube-octet-cube). These values are between 94% and 187% greater than the single-topology clusters of the same mass.

## 1. Introduction

Natural cellular materials have unique properties that are attractive to various industries, leading researchers to develop similar engineered solids for many applications within the biological and medical sciences, aviation and aerospace industries, and the automotive industry [1,2,3,4,5,6,7,8,9,10,11,12,13,14,15,16,17,18,19,20]. However, the irregular and random nature of the natural cellular materials makes it challenging to obtain predictable, repeatable properties from one specimen to another, something essential for engineering applications [21,22]. As a solution, lattice materials—with their regular, repetitive connected cells—allow for the reiterative and, consequently, controllable mechanical, thermal, and electrical properties [21,22,23]. This superiority—including characteristics such as high specific stiffness and strength as well as high energy absorption capabilities—means these periodic lattice materials are of increasing interest for a new generation of lightweight materials in numerous fields, such as weight-critical applications in aerospace and automotive sectors [24,25].

The properties of lattice materials can be controlled through the manipulation of a variety of multiscale parameters, including their relative density, the topology of the unit cell, and the material from which they are fabricated (“parent material” or “bulk material”) [25]. The advent and rapid advancement of additive manufacturing (AM) technologies have allowed for the more widespread customization and use of such materials [1,2,22,26,27,28,29]; traditional, subtractive manufacturing technologies are limited in their ability to create the necessary complex geometry, which could be on the order of micro- or nano-meters [30,31,32].

Such investigations have been plentiful in the last few decades, particularly after much of the early work on cellular materials was collected and summarized by Gibson and Ashby in their book Cellular Solids: Structure and Properties [33]. Such research has been performed analytically [34,35,36,37,38,39,40], experimentally [39,40,41,42,43,44], and numerically [39,40,42,43,45]. Andrew et al. [41] performed low-velocity experiments on polymeric plate lattices (simple cubic (SC), body-centered cubic (BCC), face-centered cubic (FCC), and combinations of those elementary cells), varying impact energy, relative density and plate thickness, cell orientation, and number of impacts. They noted that the lattices made from a combination of the elementary cells outperformed the elementary cells themselves. The hybrids also showed a direction-independent response, and the SC-BCC-FCC hybrid had a higher specific energy absorption than conventional aluminum lattices. Tancogne-Dejean et al. [43] investigated the response of octet-truss lattices under static and dynamic loading, with an interest in the role non-straight, circular cross-sectioned struts play in the energy absorption of the material. Introducing a slight taper in the struts resulted in the highest macroscopic yield strength. In their following work [46], they performed numerical simulations and experimental tests on BCC lattices with tapered struts, finding that the tapering leads to higher specific mechanical properties, including Young’s modulus and energy absorption. Özdemir et al. [44] performed quasi-static and dynamic experiments on cubic, diamond, and re-entrant cube lattices in 2016. These additively manufactured titanium alloy lattices were seen to have rate dependence based on the comparison between quasi-static and dynamic test results. Then, in 2017, they developed numerical models to predict diamond and re-entrant cube lattice behavior, using the previous experiments as validation [45]. Their finite-element models were built using beam elements for the struts of the lattices. They did not account for any imperfections that may have been presented in additively manufactured samples, yet they could accurately represent the experimental results using the numerical model. They noted that while a multi-layered re-entrant cube lattice showed rate sensitivity, a single-layer appeared rate-insensitive.

Research has also been published concerning sandwich lattice structures, in which the lattice material forms the core material, sandwiched between two thin face sheets [39,47,48]. Li et al. [39] presented theoretical, finite-element, and experimental analyses for a sandwich panel with a core of additively manufactured aluminum alloy BCC-Z lattice, noting that the three approaches produced similar results. They also concluded that the number of layers within the core significantly affected the mechanical properties of the finite panel, where the crushing strength decreased as the number of layers increased. Zhang et al. [47] manufactured sandwich panels with a hybrid core: part-polyurethane foam and part-pyramidal lattice. They found that the hybrid core samples had a greater load-carrying capacity than sandwich panels with only the polyurethane foam or only the pyramidal lattice. However, in low-velocity impact tests, the hybrid samples did not show any significant improvement in impact resistance. Mines et al. [48] investigated the impact behavior during drop-weight testing of sandwich panels with titanium alloy and stainless-steel BCC lattice cores. They observed that the stainless-steel samples were of better quality and were less sensitive to the build parameters than the titanium alloy samples but had lower specific strength. The titanium alloy samples, on the other hand, were said to be able to compete with the current aluminum honeycomb used in aerospace-grade sandwich panels.

This work presents the crashworthiness characteristics for single-layer and multi-layered lattice material, investigating the performance of single-topology and multi-topology lattice geometries, specifically with multi-layer, multi-topology (MLMT) lattices of octet-cube-octet and cube-octet-cube layers arrangement. To find the best lattice cluster design, axial compression tests were carried out, and the results were judged using crashworthiness parameters, such as energy absorption and specific energy absorption. Some crashworthiness parameters of energy absorption, and specific energy absorption values were considered to specify the best lattice design. Following this introduction, Section 2 describes the lattice design and experimental testing setup, while Section 3 defines the crashworthiness parameters used for data analysis. Section 4 presents the results and discussions from experiments, including single-layer and three-layer single-topology lattice clusters and the three-layer MLMT lattices. A conclusion and references follow Section 4.

## 2. Materials and Methods

### 2.1. Lattice Geometry, Material, and Design

**Figure 1:**
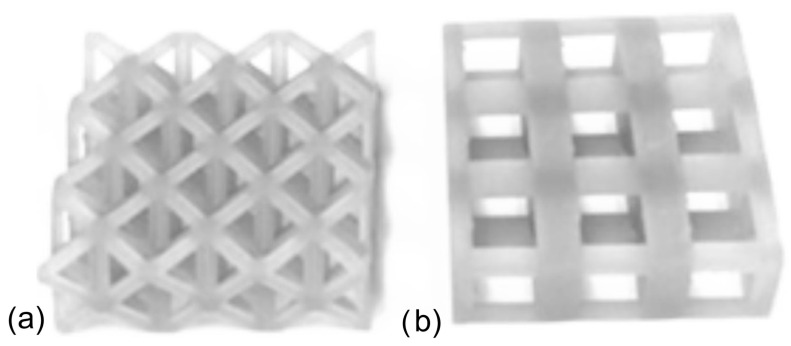
The 3 × 3 single-layer lattice samples printed with Tough 1500 resin: (**a**) octet and (**b**) cubic.

**Figure 2:**
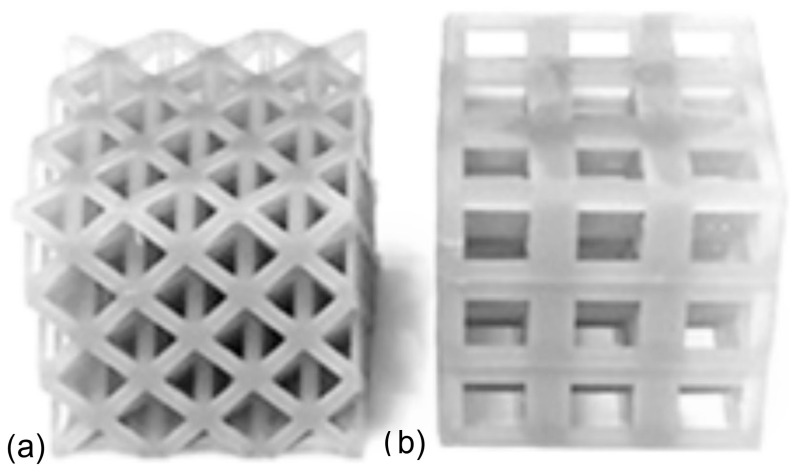
The 3 × 3 × 3 multi-layer lattice samples printed with Tough 1500 resin: (**a**) octet and (**b**) cubic.

**Figure 3:**
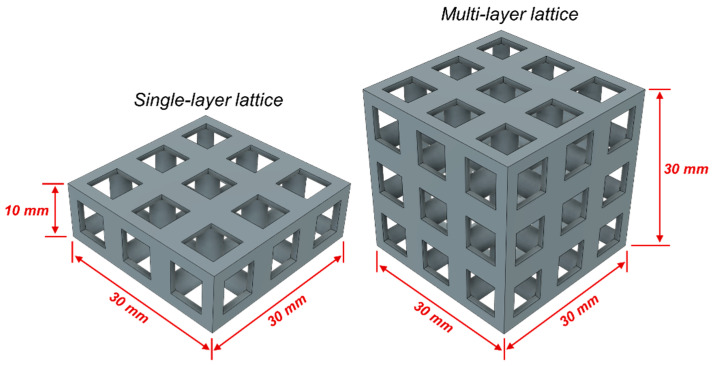
Schematic view of single- (3 × 3 × 1) and three-layer (3 × 3 × 3) lattice clusters with dimensions.

In the design of lattice structures, the constraints of uniform relative density and/or similar strut radius should be considered. In this study, cubic and octet cages were fabricated with similar relative densities: 30% for both. If the lattices were fabricated with similar strut radius, the relative density of the octet lattice would reach very high value as compared to the cubic structure (due to the fact that the mechanical behavior of this structure would start to resemble that of a solid unit cube), so this method was not preferred. Lattices were designed using cubic and/or octet truss topologies, each with an original unit cell height of 10 mm. The strut radii for each topology were 2.05 mm and 0.87 mm, respectively. Lattices had either one or three layers and three unit cells in each of the other two orthogonal directions. Sample photos of the single-topology one and three-layer lattices are provided in Figure 1 and Figure 2, respectively. A stereolithography (SLA) 3D printer (Formlab 2, ON, Canada) was used to manufacture the lattice samples. Also, an engineering resin of Though1500 was selected due to its pioneering mechanical properties, such as high strength and deformation capability. All the samples were printed solid (100% infill ratio) and with a printing height of 100 µm. The samples were washed with isopropyl alcohol (≥99%) for 10 min just after the printing process to get rid of the excessive resin. After washing the lattice samples, a UV curing process at 70 °C was applied for 60 min to enhance the mechanical properties. Also, schematic views of single- and three-layer lattice clusters with dimensions are given in Figure 3.

### 2.2. Experimental Testing Setup

**Figure 4:**
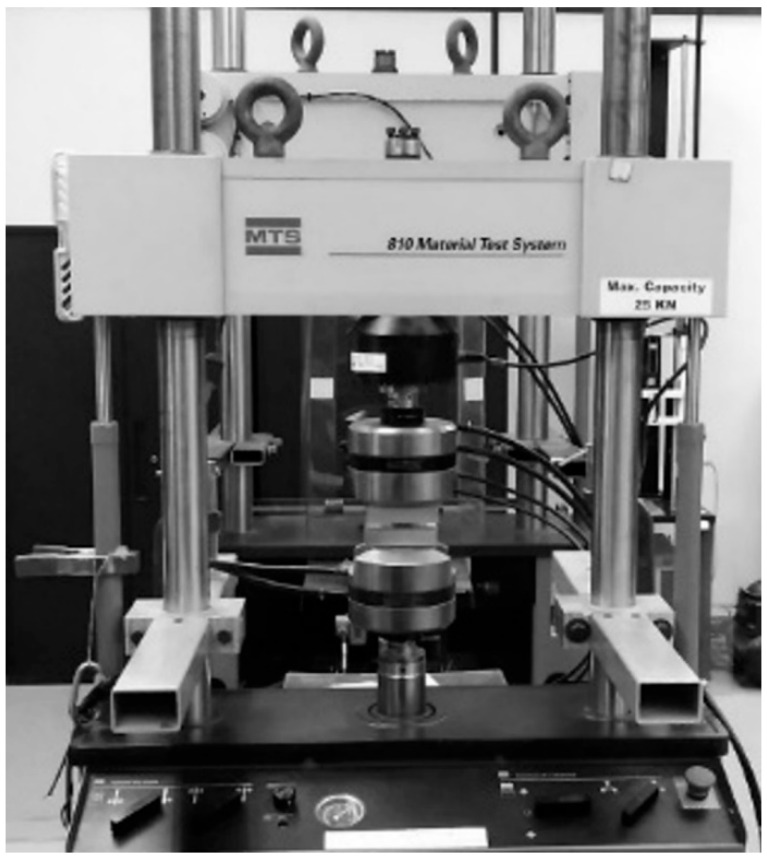
MTS machine for lattice testing.

Quasi-static compression testing was performed using an 810 MTS machine (MTS, ON, Canada.) (maximum capacity of 25 kN, Figure 4). Force-displacement data were collected directly from the machine, as the lattice samples were too small to attach an extensometer and obtain accurate data. For the first test, the single layer of 3 × 3 lattice clusters was made from a cubic or octet topology, as illustrated in Figure 1. These samples were tested at a quasi-static rate of 0.01 mm/s (strain rate of 0.001 s^−1^), and tests were repeated multiple times to ensure the accuracy of the obtained results.

**Figure 5:**
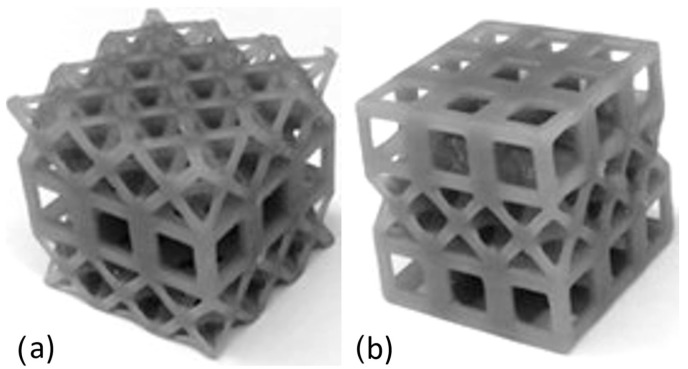
The 3 × 3 × 3 MLMT lattice samples printed with Tough 1500 resin: (**a**) octet-cube-octet and (**b**) cube-octet-cube.

For the multi-layer lattice clusters, the quasi-static test speed was set to 0.03 mm/s to keep the strain rate the same as the single-layer specimens. Photos of these samples are provided in Figure 2 for the single-topology lattices and Figure 5 for the MLMT lattices. It was noted for the printed samples of the MLMT lattices that the printing process resulted in some minimal resin build-up along the struts and around the nodes; samples with an excessive amount, deemed to have the potential to alter the performance significantly, were not utilized for experiments.

## 3. Crashworthiness Parameters for Data Analysis

Data collected from the experimental analyses included the following crashworthiness parameters, the calculation of which allows for quantifying the performance of lattice materials, in addition to stress–strain and efficiency–strain data throughout the compression event.

**Energy Absorption Efficiency.** Also, “efficiency” or η is calculated from the stress–strain (σ-ε) curve as follows:

(1) $$\eta \left(\varepsilon \right)=\frac{1}{\sigma (\varepsilon )}\int_{0}^{\varepsilon }\sigma \left(\varepsilon \right)d\varepsilon$$

where the stress is obtained from the overall compressive force divided by the surface area of the upper surface of the lattice unit cell envelope (not the surface area of the lattice structures that the upper plate touches), and the strain is obtained from the displacement of the uppermost surface of the lattice divided by the original lattice unit cell height, a method utilized in [49,50,51].

**Densification Strain.** Per the energy absorption efficiency method, the densification strain, ε_D_, is as follows:

(2) $${\left.\frac{d\eta (\varepsilon )}{d\varepsilon }\right|}_{\varepsilon =\varepsilon_{D}}=0$$

which is the strain at the maximum efficiency point [49,52,53].

**Plateau Stress.** The plateau stress, σ_pl_, along with densification strain are considered to be the most important values with regard to the energy absorption of materials [52]. This parameter is calculated as follows [49]:

(3) $$\sigma_{\mathrm{pl}}=\frac{\int_{0}^{\varepsilon_{D}}\sigma \left(\varepsilon \right)d\varepsilon }{\varepsilon_{D}}$$

**Energy Absorption.** Energy absorption, i.e., EA or IE (internal energy), is calculated as given below:

(4) $$\mathrm{EA}=V\int_{0}^{\varepsilon_{D}}\sigma \left(\varepsilon \right)d\varepsilon$$

It should be noted that while Equation (4) utilizes the densification strain as the final integration point, the selected strain value within this integral does vary across the literature [43,49,54,55,56,57].

**Specific Energy Absorption.** Specific energy absorption (SEA) can be calculated either per unit volume—SEA_V_—or per unit mass—SEA_m_. Equations for both of these SEAs are provided as follows [49,56,58]:

(5) $${\mathrm{SEA}}_{V}=\frac{\mathrm{EA}}{V}=\int_{0}^{\varepsilon_{D}}\sigma \left(\varepsilon \right)d\varepsilon ,$$

(6) $${\mathrm{SEA}}_{m}=\frac{\mathrm{EA}}{m}=\frac{\int_{0}^{\varepsilon_{D}}\sigma \left(\varepsilon \right)d\varepsilon }{\bar{\rho }\rho_{0}},$$

where V and m are the volume and the mass of the lattice structure, respectively. It should be noted that these values are also calculated up to the densification strain, as with EA.

## 4. Results and Discussion

### 4.1. Single-Layer Lattice Clusters

**Figure 6:**
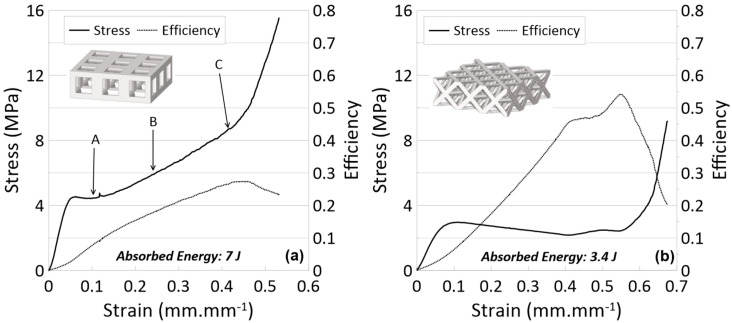
Stress vs. strain and efficiency vs. strain curves for experiments of (**a**) cubic and (**b**) octet lattice printed with Tough 1500 resin. See Figure 7 for photos relating to letter labels.

**Figure 7:**
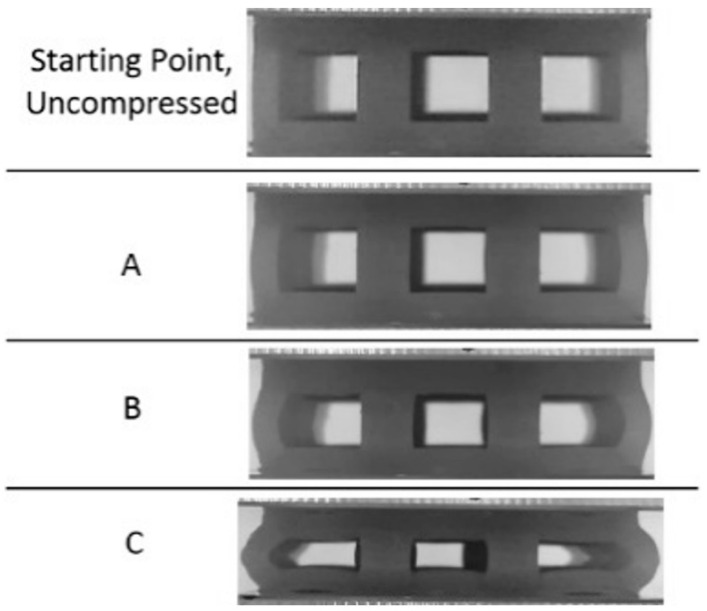
Progressive compression of experimental sample and numerical model of single-layer cube lattice printed with Tough 1500 resin.

All the experiments were repeated at least three times for each combination. However, only the curve representing the mean value is given in the graphs to avoid misunderstanding. Stress–strain and efficiency–strain results are presented in Figure 6, with Figure 7 providing images corresponding to the labeled points in the stress–strain figures. Table 1 (at the end of Section 4.3) summarizes numerical data for the crashworthiness parameters for all experiments completed.

Looking first at the cubic topology in Figure 6a, the elastic region continues until the stress surpasses 4 MPa at a strain of approximately 0.05 mm/mm. After this point, there is a very brief plateau, but the stress continues to increase gradually until the densification point of 0.45 mm/mm, where the stress sharply increases. The average stress between the yield point and densification was calculated at 5.8 MPa. Observing the snapshots of the compression event in Figure 7 for the cubic topology, it can be seen that the deformation behavior of the single-layer lattice is buckling-dominated: At the beginning, the outer struts of the cubic lattice bow outwards, hinging at the upper and lower nodes, which contact two compression plates. Next, looking at the octet topology in Figure 6b, the elastic region continues until reaching a slightly larger strain—just below 0.1 mm/mm—but does not reach the same maximum as the cubic topology. Once in the plateau region, the stress remains more constant than compared to the cubic topology but does gradually decrease, leading up to the densification strain at about 0.5 mm/mm. While the sustained stress of the octet lattice is not as large as the cubic lattice, the maximum efficiency is greater at 0.55 compared to 0.27.

### 4.2. Multi-Layer Lattice Clusters

**Figure 8:**
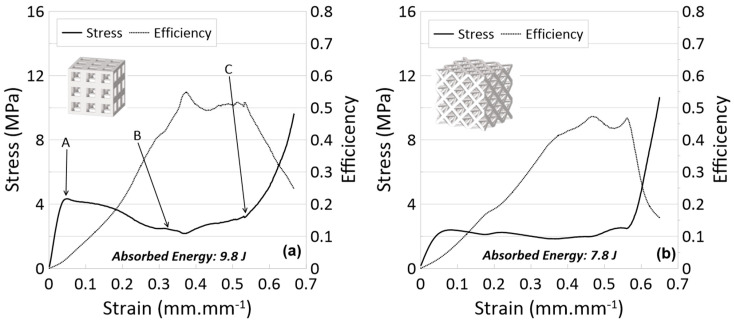
Stress vs. strain and efficiency vs. strain curves for experiments of (**a**) cubic and (**b**) octet multi-layer lattices printed with Tough 1500 resin. See Figure 8 for photos relating to letter labels.

**Figure 9:**
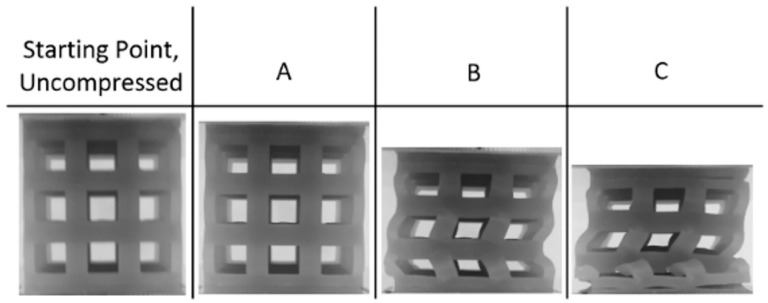
Progressive compression of the multi-layer cube lattice sample printed with Tough 1500 resin.

Stress–strain and efficiency–strain results are presented in Figure 8, while Figure 9 illustrates images from the compression event. Table 1 (at the end of Section 4.3) summarizes the crashworthiness parameters for all experiments in this work.

First, when looking at the results from the cubic lattice (Figure 8a), it can be seen that there are similarities with the single-layer cubic results, particularly within the elastic region; the elastic region surpasses a stress of 4 MPa and ends at a strain of about 0.05 mm/mm. However, the plateau region of the multi-layer lattice differs from the single-layer cubic lattice. Within this region, the stress sustained by the multi-layer lattice barely plateaus before beginning to decrease, reaching a minimum just before a strain of 0.4 mm/mm. After this point, the stress continually increases. Based on the deformation images in Figure 9, it can be noted that the lattice had a buckling deformation at the beginning of the test (point A in Figure 9) similar to that of the single-layer cubic lattice cluster. However, a dramatic buckling and collapse behavior occurred in the multi-layer cubic lattice in contrast to the single-layer cubic lattice (especially noticeable in Figure 9, point C). This can be explained as an effect of movable nodes between the upper and lower faces in the multi-layer cubic lattice. This dramatic buckling deformation is also the reason behind the decrease in stress after the yield point; the layer had collapsed. Since there are no movable nodes in a single-layer cubic lattice, a pure buckling deformation occurred. Additionally, no decrease was observed in the stress curve (Figure 8a).

Then, when looking at the octet lattice results (Figure 8b), there are again similarities with its single-layer variant. Within the plateau region, after a yield stress of just over 2 MPa at a strain of almost 0.1 mm/mm, the stress stays steady, giving a plateau stress of 2.2 MPa. While the single-layer octet does show a stress decrease within this region, the three-layer lattice generally does not. At the end of the plateau region, there is a noticeable, sharp increase in stress at densification. A comparison of the two lattice topologies shows, again, that the cubic lattice withstands higher stresses for a given strain than the octet lattice. Consequently, by densification, the octet lattice absorbed less energy (average of 7.8 J) than the cubic lattice (average of 9.8 J). While the single-layer cubic lattice had a lower efficiency than the single-layer octet lattice, with the three-layer lattices, the difference between efficiencies decreased, and the cubic lattice efficiency was actually larger than that of the octet lattice (0.59 versus 0.46, respectively).

### 4.3. Multi-Topology Lattice (Multi-Layer) Clusters

**Figure 10:**
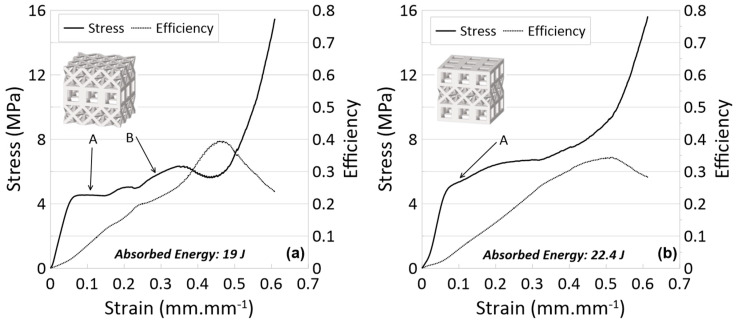
Stress vs. strain and efficiency vs. strain curves for experiments of MLMT lattices: (**a**) octet-cube-octet and (**b**) cube-octet-cube printed with Tough 1500 resin. Refer to Figure 10 for photos relating to letter labels.

**Figure 11:**
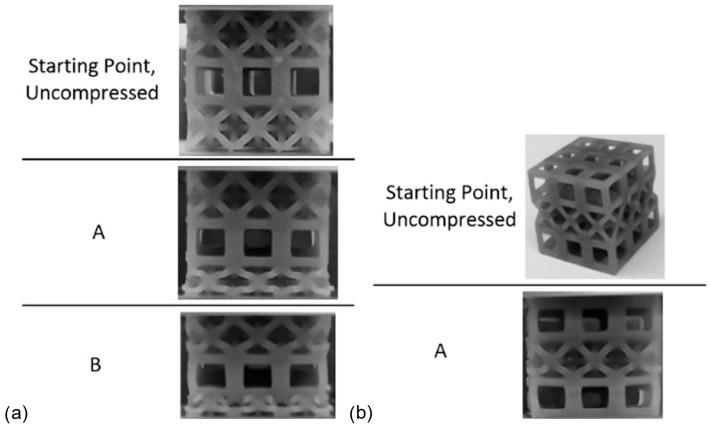
Progressive compression of the sample of MLMT lattices printed with Tough 1500 resin: (**a**) octet-cube-octet and (**b**) cube-octet-cube.

The stress–strain and efficiency–strain results are provided in Figure 10 for the MLMT lattices and snapshots of the compression event for the cube-octet-cube (C-O-C) and octet-cube-octet (O-C-O) lattices are provided in Figure 11. Table 1 summarizes the numerical data for the crashworthiness parameters for all lattices investigated.

For the O-C-O lattice (Figure 10a), the elastic region ends at a stress just above 4 MPa and plateaus only for a short strain before generally increasing in value with an increase in strain. The stress sharply increases at a density of 0.49 mm/mm. For the C-O-C lattice (Figure 10b), stress–strain results are similar; the elastic region ends at a stress of about 5 MPa, and the densification strain is 0.48 mm/mm. In comparing efficiency results, the O-C-O lattice maximum is greater than the C-O-C lattice (0.36 versus 0.31, respectively). In considering these results in comparison to their component parts —octet and cubic layers—the slightly higher yield stress for the C-O-C lattice can be drawn from the presence of two cubic layers (which were previously seen to have higher stress values than the octet layers). This same knowledge can be used to understand why the C-O-C lattice, within the plateau region, also has slightly higher stress values. Indeed, the plateau stress of the C-O-C lattice is 5.8 MPa versus the 4.9 MPa plateau stress of the O-C-O lattice. Interestingly, the efficiency of the O-C-O lattice is higher than the efficiency of the C-O-C lattice, though in looking at the results for the single-layer lattices (and not those results from the three-layer lattices), it could perhaps be concluded that, in this configuration, the cubic layers understandably act as single layers, and with the octet layer sandwiched in the middle, they do not act in unison to lead to an increase in efficiency (which was seen in the three-layer cubic in comparison to the three-layer octet). Looking at the four variations of three-layer lattices, it is possible to easily conclude that the multi-layer lattices (maintaining the same relative density) have higher energy absorption capabilities than the single-topology lattices. In experiments, the multi-layer cubic and octet lattices had energy absorptions of 9.8 J and 7.8 J, respectively, while the MLMT lattices had approximately two or more times the energy absorbed up to densification. Even by summing the single-layer EAs for each MLMT lattice, the actual EA of the MLMT lattice was greater (O-C-O) at 13.8 J from single-layer additions versus 19.0 J experimentally (C-O-C 17.4 J versus 22.4 J). It is also possible to see the influence of the different topology layers on the overall performance of the MLMT lattice.

It is seen from Table 1 that the absorbed energy of the samples varies in a range related to the layer numbers and lattice topology. For instance, the octet lattice cluster absorbs almost two times more energy when using three layers instead of a single layer. However, the three-layered cubic lattice could not reach the same increase compared to that of the single-layer cubic lattice. This could be explained by the dramatic buckling deformation behavior in multi-layer lattice cubic clusters (Figure 9). Additionally, using different lattice topologies together resulted in an increase in absorbed energy. The C-O-C sample absorbed the highest energy compared to the other clusters. It is also important to show that placing an octet lattice layer between cubic layers enhanced the structural stability.

## 5. Conclusions

The quasi-static compression of additively manufactured single-layer and multi-layer lattice specimens was completed. Utilizing force and displacement data, crashworthiness parameters including maximum efficiency, densification strain, plateau stress, and energy absorption were calculated for all experiments. These parameters as well as stress–strain, efficiency–strain, and deformation behavior snapshots, were then compared and discussed. The performance of multi-topology multi-layer lattices was also investigated and discussed. Some key conclusions from this work with the Tough 1500 resin include the following:

- The print quality of the resin lattices was generally good, with minimal build-up of resin on the struts and around the nodes;
- Observations of the experiments showed the buckling-dominated response of the cubic lattice and the stretching-dominated response of the octet lattice. The cubic lattices carried higher stresses than the octet lattices for a given strain and also had higher plateau stresses and energy absorptions, though the octet lattices had higher maximum efficiencies;
- In creating multi-layer lattices with multiple topologies, the energy absorption capability was increased beyond the capacity of the multi-layer, single-topology lattices on their own. All work was performed with lattices at a relative density of 30%, meaning that the increase in energy absorption in the MLMT lattices was also an overall increase in specific energy absorption per mass by these unique lattices.

## Figures and Tables

**Table 1:** Summary of crashworthiness parameters for all lattices.

Size	Topology ^1^	Max. Eff. ^2^	Dens. Strain ^2^ [mm/mm]	EA ^2^ [J]	${SEA}_{V}$ ^2^ [×10^6^ J/m^3^]	${SEA}_{m}$ ^2^ [×10^3^ J/kg]	Plat. Stress ^2^ [MPa]
3 × 3 × 1	C	0.27	0.46	7.0	2.61	2.44	5.8
		0.27	0.45	7.0	2.61	2.44	5.8
		0.25	0.45	6.9	2.60	2.43	5.8
	O	0.56	0.51	3.5	1.26	1.16	2.5
		0.55	0.50	3.4	1.25	1.16	2.5
		0.55	0.50	3.4	1.25	1.16	2.5
3 × 3 × 3	C	0.58	0.38	9.6	1.20	1.12	3.1
		0.59	0.39	9.8	1.21	1.13	3.1
		0.59	0.39	9.9	1.22	1.13	3.1
	O	0.46	0.46	7.9	0.97	0.90	2.2
		0.46	0.45	7.8	0.97	0.90	2.2
		0.46	0.45	7.8	0.97	0.90	2.2
3 × 3 × 3 MLMT	O-C-O	0.36	0.49	19.1	2.34	2.19	4.9
		0.36	0.49	19.0	2.34	2.19	4.9
		0.35	0.47	18.8	2.32	2.18	4.9
	C-O-C	0.31	0.46	22.1	2.75	2.58	5.8
		0.31	0.48	22.4	2.77	2.59	5.8
		0.32	0.49	22.6	2.79	2.61	5.8

^1^ C = cube; O = octet. ^2^ Only average values are summarized in this table.

## Data Availability

The original contributions presented in the study are included in the article, and further inquiries can be directed to the corresponding authors.
